# Selection and promotion processes are not associated by the relative age effect in an elite Spanish soccer academy

**DOI:** 10.1371/journal.pone.0219945

**Published:** 2019-07-24

**Authors:** Daniel Castillo, Benito Pérez-González, Javier Raya-González, Álvaro Fernández-Luna, Pablo Burillo, Ángel Lago-Rodríguez

**Affiliations:** 1 Universidad Isabel I. Faculty of Health Sciences, Burgos, Spain; 2 Universidad Europea de Madrid, Faculty of Sports Sciences, Madrid, Spain; Instituto Politecnico de Viana do Castelo, PORTUGAL

## Abstract

The aim of this study was to analyze the influence of the relative age effect (RAE) on the selection and promotion processes in an elite soccer academy. One hundred and eleven elite youth players who belonged to an elite soccer club from the Spanish “La Liga” participated in this study. Players were classified into three age-categories: under 14 years (U14), under 16 years (U16) and under 18 years (U18); and they were also classified in quartiles based on their date of birth (i.e., January-March, Q1; April-June, Q2; July-September, Q3; October-December, Q4). In addition, two further classification criteria were established based on the selection (i.e., selected and non-selected players) and promotion (i.e., promoted and non-promoted players) processes. The main results showed that in U14 and U16 age-categories, players born early in the year were over-represented compared to players born late in the year, although birth-distribution was not associated with the likelihood of a player to be selected or promoted. In addition, less fat in sum skinfolds, less percentage of fat, higher percentage of muscle and lower endomorphy and mesomorphy components were showed in U14 selected players, in comparison with non-selected players. Likewise, better sprint performance was found in U16 selected players versus non-selected ones. However, no significant differences on anthropometry, body composition, somatotype and physical performance were found between promoted and non-promoted players. Therefore, our results suggest there is need for coaches to reorient their talent identification programs in order to make sure that players selected to continue playing in the club have the potential to promote to the excellence in soccer.

## Introduction

In order to ensure fair competition between players with similar levels of development and growth, youth soccer competitions are arranged by age-groups [1]. However, this competition system does not allow to completely avoid players with different chronological age competing within the same category. Since the cut-off for players distribution within a specific category is determined by year onset (1^st^ January), and players born in two different years may be competing at the same age-category, the chronological age difference between young competitors may be up to 24 months [2]. This variance leads to considerable anthropometry and physical conditioning differences between players competing against each other [3–5]. So, this phenomenon should be taken with special attention in the identification of talented athletes during early age.

It has been extensively reported in the literature that these growth-related biological differences among players of the same team and category determine the chances of a player to be included in the talent identification programs undergone in elite soccer academies [6,7]. Players born at the beginning of the year are more likely to be selected to be part of an elite soccer academy, compared to those born at the end of the same year [8]. This phenomenon is known as relative age effect (RAE) [1], and it has been well-documented in soccer for the past two decades [2,9–15]. However, there is a lack of knowledge regarding the association between RAE and the selection and promotion of elite young athletes. Therefore, it would be interesting to investigate the influence of this phenomenon on the selection and promotion processes undergone by elite soccer academies.

Determining the main traits which can influence the specific competence of soccer players is far from perfect [16], because soccer is a multifactorial sport in which success is conditioned by the interaction of multiple relevant features such as anthropometric, body composition, somatotype, physical and physiological factors, as well as soccer-specific skills [17]. In this sense, it is well-known that making predictions about future performance at early ages (i.e., U10-U18 age-categories) is a complex task [16], although elite soccer academies are expected to find the reason determining the chances of a player to succeed in the club or to achieve high competitive levels [18]. Therefore, further information is needed in order to improve the talent identification process, and thus stablishing the specific characteristics of talented players who progress in an elite soccer academy is crucial for coaches, in order to optimize talent development programs.

Previous investigations have been carried out in order to optimize the selection process, defined as the progression to the next age-categories and standard of play of talented youth soccer players [9,19,20]. In this respect, it has been observed that athletes selected to continue playing for a Spanish soccer academy during the next age-category had lower weight, higher height, lower percentage of fat and were better in agility test, when compared to non-selected players [9]. Besides, Gil et al. [20] observed that players pre-selected to entry in an elite soccer academy displayed better agility and endurance compared to non-selected players. Furthermore, it has been reported that the selection criteria depend on the age-category of the talented players. Whereas physical performance was crucial for players from U13 age-category in order to be selected to continue playing for the soccer academy, neither physical conditioning nor anthropometry and body composition characteristics were vital for players from U15 age-category in order to progress to the following stage of the club [19]. Since there is no consensus about the main characteristics which seem to ascertain the continuity of soccer players in a club, further studies are needed in order to understand the selection process undergone by youth soccer academies.

Several attempts have been made to evaluate the relevance of the anthropometry, physical fitness and soccer-related characteristics of youth players when trying to explain whether they achieved a professional status or remained amateur [3,17,21]. In this respect, Deprez et al. [11] observed that anthropometry values were not useful to discriminate between players who achieved a professional contract and those who did not. Conversely, Carling et al. [21] observed that players who achieved professional status presented lower weight and body fat at the age of 13 years old, when compared to players who remained amateur. In this way, Le Gall et al. [22] suggested that anthropometric and fitness assessments of elite youth male soccer players may play an important role in determining their chances of proceeding to higher standard of play. Regarding the physical performance characteristics, Deprez et al. [11] showed that players who signed a professional contract had higher jump performance and were faster than player who did not achieve a professional status. In addition, Gonaus and Muller [23] reported better sprint and jump performance in those players who subsequently had been drafted into a youth national team. Otherwise, several investigations showed that aerobic endurance and sprint and jump performance do not determine the professional career of youth soccer players [17,24]. The aforementioned studies analyzed whether some anthropometric and physical characteristics of soccer players could determine their promotion to the professionalism. However, it would also be interesting to look into whether players achieve the top category or standard of play of their specific age-category.

Considering that a wide knowledge is needed regarding talent identification in youth elite soccer players, the aim of this study was to analyze the influence of the relative age effect (RAE) and physical conditioning features (i.e., anthropometry, body composition, somatotype, and physical performance) on the selection and promotion processes in an elite soccer academy.

## Materials and methods

### Participants

One hundred and eleven elite youth male outfield players (age = 16.3 ± 2.1 years; height = 170.1 ± 7.9, body mass = 62.17 ± 7.60 kg) who belonged to a soccer academy from the Spanish “La Liga” international club participated in this study. All players trained at least four times per week (1.5 h training per day) and were involved in a competitive match over the weekend. Players who had been injured during the two months preceding the testing sessions were excluded from participating in this study. This study was carried out from the 2013–14 until 2018–19 season, since in this last competitive season performance data were also recorded. Players were classified into three age-categories: under 14 years (U14), under 16 years (U16) and under 18 years (U18). In addition, players were further categorized based on other two criteria: selection and promotion processes [9]. For each age-category, those players who continued playing in the same Club during the following season were categorized as *selected*, whereas players who did not continue playing in the Club were categorized as *non-selected*. Furthermore, players whose sport career was to compete at higher standard of play in their age (i.e., U18 National Youth League or 2°B Division) until the 2018/2019 season were classified as *promoted*, whereas players whose sport career was not to compete at their higher standard of play in their age until the 2018/2019 season were classified as *non-promoted*. In order to carry out this classification, researchers and the technical director of the Club found the players’ highest competitive level until the end of 2018/2019 season. The Club authorized the use of a dataset for research purposes wherever anonymity was ensured. Besides, the Club had the permission of parents or legal guardians, who signed the written consent, to perform the physical test to the players. This investigation was performed in accordance to the Declaration of Helsinki (2013), was approved by the Ethics Committee of the University of Camilo José Cela (UCJC), and met the ethical standards in Sport and Exercise Science Research [25].

### Procedure

A descriptive study was carried out during the 2013/2014 competitive season. First, the birth date of the 111 youth players who participated in this study was recorded with the purpose of identifying the impact of RAE in an elite soccer academy. The birth-date distribution of the players was analyzed by quarters (Q1 = 1^st^ January to 31^st^ March; Q2 = 1^st^ April to 30^th^ June; Q3 = 1^st^ July to 30^th^ September; Q4 = 1^st^ October to 31^st^ December). Second, anthropometry (i.e., body mass, height, six skinfolds, thigh length and biestyloid at the wrist), body composition (i.e., percentage of fat and muscle), somatotype (i.e., endomorphy, mesomorphy and ectomorphy) and physical performance (i.e., two jump tests [abalakov and abalakov with dominant leg], 10 and 30 m sprint tests and flexibility) of the participants were assessed for the purpose of analyzing the selection and promotion processes. This data collection session was undertaken in April, near to the end of the competitive season, and three days after the preceding competitive match, allowing physical recovery. Players were given advice about a similar diet (55% calories were derived from carbohydrate, 25% from fat and 20% from protein) and were instructed to avoid caffeine-containing products during 72 h before the testing sessions [26].

### Anthropometry, body composition and somatotype

Body mass (kg) and height (cm) were measured using a balance and stadiometer (Seca 285, Bonn, Germany), respectively. Six skinfold thickness (mm) (i.e., triceps, subscapular, abdominal, suprailial, thigh and lower leg) using a skinfold caliper (Holtain, Crymych, United Kingdom) were registered. The sum of the six measurements was used for further analysis. Also, the thigh length (cm) and the biestyloid at the wrist (cm) were measured with an anthropometric tape measure (Holtain 110P-98606, Crymych, United Kingdom) and a caliper (HLT-100, Holtain Ltd., Crymych, United Kingdom), respectively. Anthropometric parameters were measured by a certified anthropometrist, according to the International Society for the Advancement of Kinanthropometry (ISAK) [27]. In addition, body fat and muscle percentages were estimated using segmental bioimpedance (BC-418, Tanita, Japan) following appropriate standardizations and using the standard built-in prediction equations for children [28]. Finally, three components of somatotype (i.e., endomorphy, mesomorphy and ectomorphy) were also calculated by using the Heath-Carter somatochart [29].

### Physical performance

Prior to data collection, players undertook a 15 min standardized warm-up, consisting of 6 min of slow jogging and strolling locomotion followed by 5 min progressive sprints and accelerations, finishing with 4 min of jumps. Each player undertook, in this order, two jump tests (i.e., abalakov and abalakov with dominant leg), a 30 m sprint tests and a flexibility test, which were interspersed with 5 min of semi-active rest.

#### Vertical jump performance

Players performed two maximal bilateral countermovement jumps with arms (Abalakov) and two maximal unilateral countermovement jumps with arms with dominant leg (D-Abalakov) on a force platform sampling at 500 Hz (Quattro Jump; Kistler, Winterthur, Switzerland), separated by a rest period of approximately 10 s and 90 s between tests [30]. Players started from a standing position, swinging their arms and bending their knees, and jumped as high as possible with arm swing. The highest jump was used for further analysis. Jump height was calculated from the flight time by means of the following formula: h = gt^2^/8 [31]. Coefficient of variation (CV) for the jump tests was calculated (Abalakov: 7.2 ± 5.7%, D-Abalakov 7.5 ± 6.7%).

#### 10 and 30 m sprint performance

Players performed two sprint trials of 30 m length, with 120 s rest period between each one [30]. Players’ starting position was 0.5 m away from the starting point, and run-off when they felt ready. Time recording was automatically activated as participants passed the first gate (Microgate Polifemo, Bolzano, Italy), that is, at 0 m line. Split times were recorded at 10 m and 30 m. The best score was then used for analysis. CV for the sprint tests was calculated (10 m sprint: 1.1 ± 1.1%, 30 m sprint: 1.0 ± 0.5%). This test was performed on a grass soccer pitch (i.e., 17–22°C, 60–70% humidity) and players wore their own soccer boots.

#### Flexibility

The lower back and hamstrings flexibility was measured using the sit and reach test. Players started sitting on the floor with the knees straight, legs together, and the soles of the feet positioned flat against a box and performed the test as described by Muyor, Vaquero-Cristobal, Alacid, and Lopez-Minarro [32]. Two trials were performed, and the best one was used for further analysis. CV was 1.81± 0.6%.

### Statistical analysis

Data are presented as means ± standard deviations (SD). The normal distribution of results of the variables registered was tested using the Kolmogorov-Smirnov test, and the equality of the variances was established with Levene’s test. The observed and expected birth distribution for each age-category (U14, U16, and U18) and the overall dataset were compared using separate Chi-square (*χ*^2^) tests. Percentage of birth for each quartile and age-category in Spain was used as the expected distribution (data extracted from Instituto Nacional de Estadística database). In addition, odds ratios (ORs) and 95% confidence intervals (95% CI) were calculated for the first, second, and third quartile, relative to the last quartile of the year. Due to the low statistical power previously attributed to the Chi-square (*χ*^2^) test when analysing the effect of RAE, and its vulnerability to non-RAE signature [33], the Poisson regression for analyzing low count data has been suggested as an alternative [34]. Thus, indications from previous work were followed [35], and the week of born (W_B_) for each player was computed. Players born between 1^st^ and 7^th^ January were assigned to W_B__1, players born between 8^th^ and 14^th^ January were categorized as W_B__2 and so on. We then calculated the time of birth (t_B_) according to the formula *t_B_* = (*W_B_*−0,5)/52. Therefore, t_B_ measures how far from the beginning of the year a player was born, and it ranges between 0 and 1. This calculations allowed to perform a Poisson regression to count data for the overall dataset, in order to calculate how the frequency of birth in a given week (*W_B_*) was explained by the time of birth (t_B_), using the formula $W_{B}=e^{\left(b0+b1t_{B}\right)}$. In addition, the Index of Discrimination (ID) was computed as *e*^−*b*0^, which provides the relative odds of being selected for a player born on day 1 compared to a player born in day 365 of the competition year [34]. In order to compare the birth-date distribution between selected versus non-selected players and promoted versus non-promoted players, separate Chi-square tests were performed. When an expected frequency lower than 5 was observed in a contingency table, Fisher’s exact test was computed and reported. In addition, separate Student’s t-test for independent samples were used to compare results on anthropometry, body composition and physical fitness between selected versus non-selected players and promoted versus non-promoted players independently for each category and for all players. Practical significance was assessed by calculating Cohen’s *d* effect size [36] and was considered as large (>0.8), moderate (0.8–0.5), small (0.5–0.2) and trivial (<0.02) [37]. Statistical analysis was performed using the Statistical Package for Social Sciences (version 21.0 for Windows, SPSS Inc, Chicago, IL, USA). Statistical significance was set at p < 0.05.

## Results and discussion

### Relative age effect (RAE)

The birth-date distributions for the three age-categories analyzed (U14, U16, and U18), and for the overall sample (Total) is summarized in Table 1. The chi-square test revealed that the birth-date distribution differed from the general Spanish population for U14, U16, and Total (U14, *χ*^2^ = 21.242, p < 0.001; U16, *χ*^2^ = 23.442, p < 0.001; Total, *χ*^2^ = 48.119, p < 0.001). The birth-date distribution for the U18 age-category was not significantly different from the observed in the population, although it should be noted that it was very close to significance (*χ*^2^ = 7.792, p = 0.052).

**Table 1:** Percentage of players born in each of the four quarters (Q) of the year along each category. Odds ratios (OR) (and 95% confidence intervals) are shown, examining birth-date distributions in relation to Q4 for each age-category (U14, U16, U18) and overall (Total).

	U14	U16	U18	Total
**Q1**	52.6	50.0	40.0	47.7
**Q2**	26.3	36.8	31.4	31.5
**Q3**	18.4	7.9	17.1	14.4
**Q4**	2.6	5.3	11.4	6.3
*χ*^2^	21.242	23.442	7.792	48.119
**df**	3	3	3	3
**p**	<0.001	<0.001	0.052	<0.001
**OR**_**Q1/Q4**_	20.23 (5.82–70.27)	9.43 (3.85–23.09)	3.51 (1.82–6.78)	7.57 (3.3–17.38)
**OR**_**Q2/Q4**_	10.12 (2.83–36.19)	6.94 (2.79–17.25)	2.75 (1.4–5.42)	5 (2.13–11.76)
**OR**_**Q3/Q4**_	7.08 (1.93–25.94)	1.49 (0.5–4.48)	1.5 (0.71–3.17)	2.29 (0.9–5.84)

Abbreviations: df: degrees of freedom; p: level of signification.

**Fig 1:**
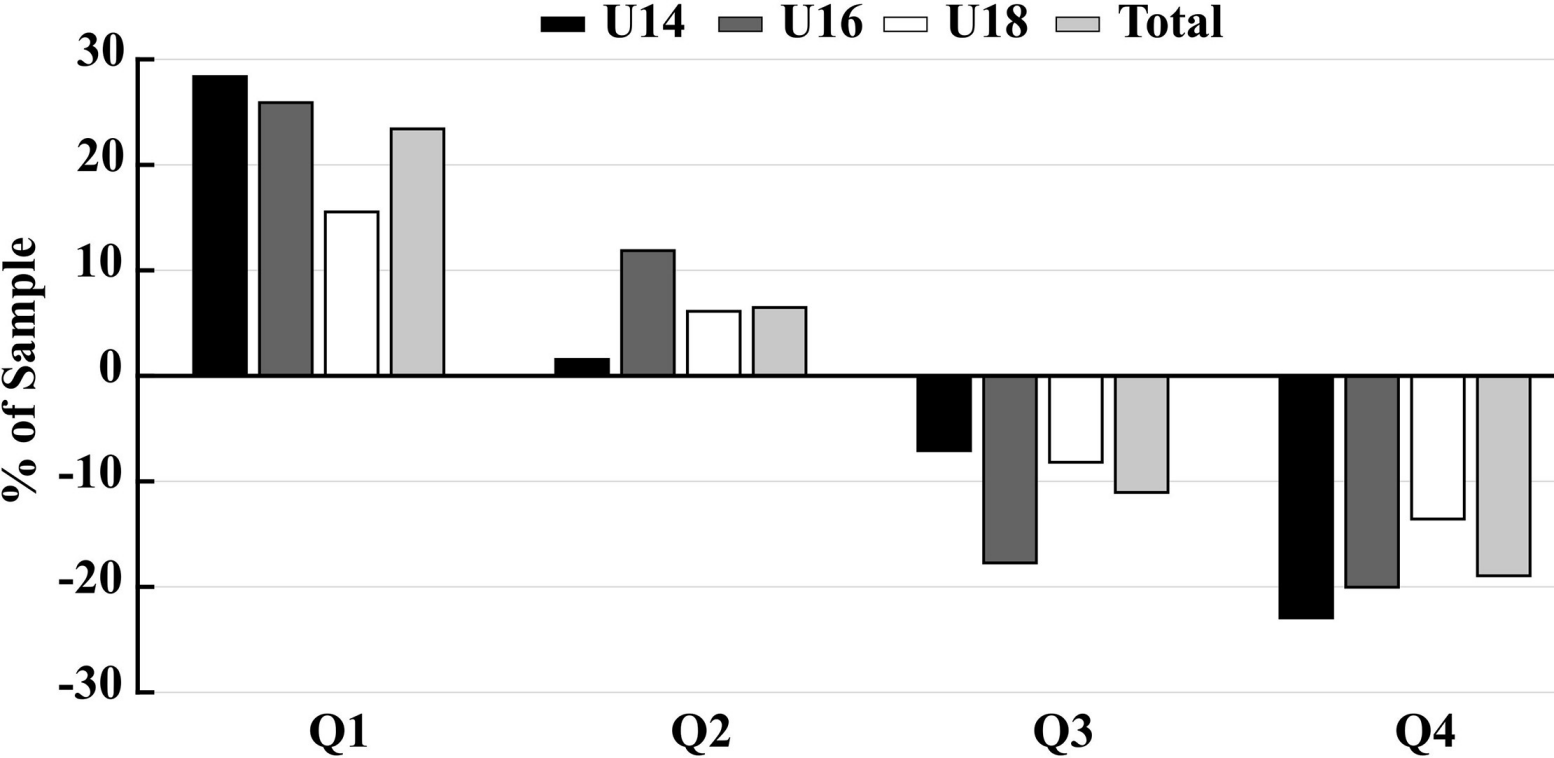
Differences in the birth-date distributions between the general population and our sample is shown in this figure. The zero value corresponds to the Spanish population’s birth-date distribution for each age-category, and overall. Thus, positive values indicate over-representation of players born at the quarter, whereas negative values indicate under-representation of players born at the quarter, compared to the population’s birth-date distribution. Abbreviations: U14 = under 14 age-category; U16 = under 16 age-category; U18 = under 18 age-category; Q = quarter of the year.

Table 1 also displays results found when the odds ratios and 95% CI were computed. Overall, the odds of playing for an elite soccer academy were 7.57 higher for players born at year onset (Q1) than for players born at the end of the competition year (Q4). Moreover, these odds decreased as the relative age difference diminished (OR_Q2/Q4_ = 5; OR_Q3/Q4_ = 2.29). These results were mirrored for the three age-categories (i.e., U14, U16, and U18). Interestingly, between-group comparisons revealed that the effect of RAE decreased as the age-category increased, since the odds of playing for an elite soccer team for players born at Q1 were larger at U14 category, compared with U16 and U18 (OR: 20.23; 9.43; 3.51, respectively). Fig 1 represents the differences in the birth-date distributions between our sample and the population.

**Fig 2:**
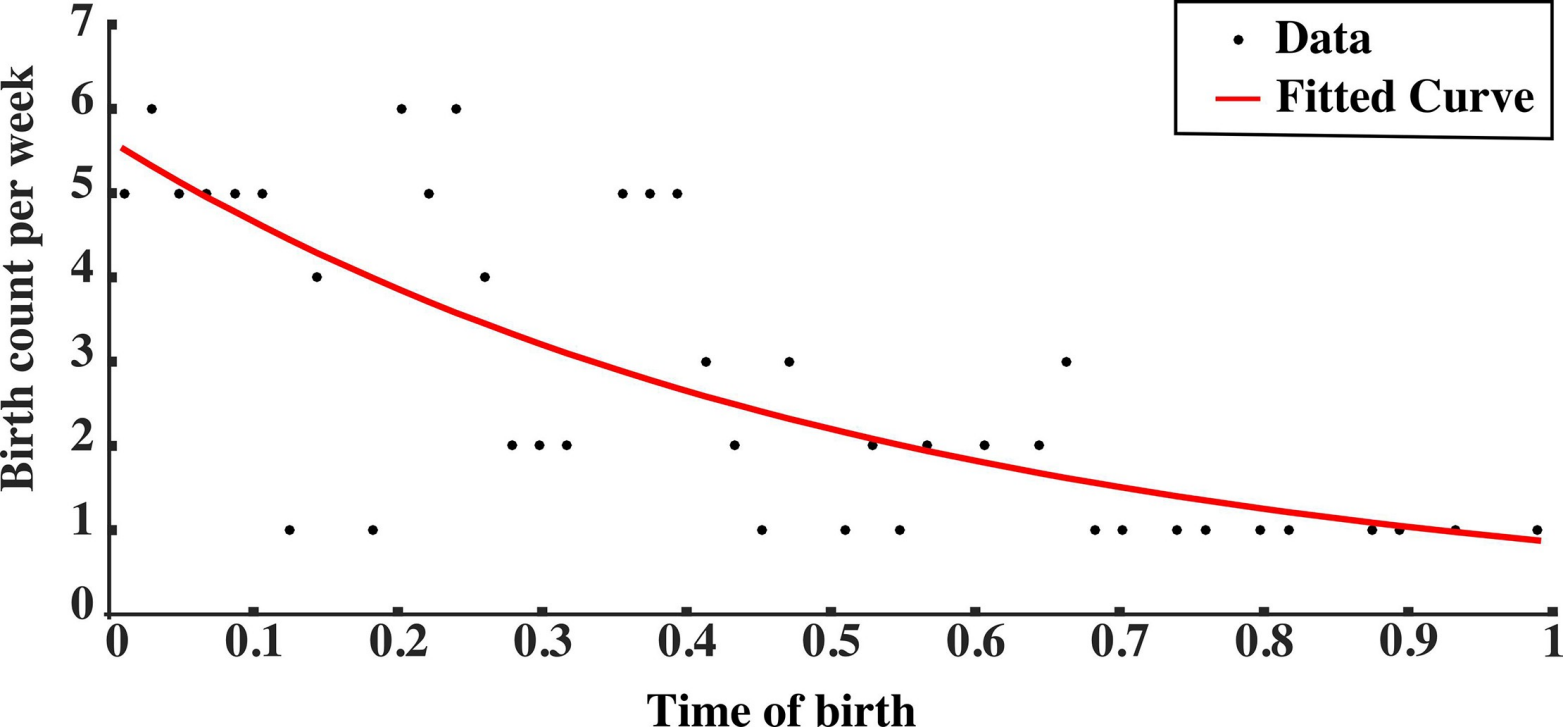
Scatterplot for RAE frequency by week. The black line represents the best fit of the Poisson regression.

Results of the Poisson Regression analysis for the overall dataset were in line with results obtained for the Chi-square analysis. A summary of the results is shown in Table 2, and the scatterplot of relative birth frequency by week is reported in Fig 2. The frequency of birth was larger at year onset, compared to the end of the year. The index of discrimination (ID) showed that players born at the beginning of the competition year were 6.55 more likely to be part of the youth soccer elite academy.

**Table 2:** Relative Age Effect (RAE) according to the Poisson regression analysis for the overall sample.

Poisson Regression's Parameters						
W_B_	t_B_	b_0_	b_1_	ID	R^2^	p
16.8 ± 12.5	0.31 ± 0.24	1.727	-1.879	6.55	0.28	<0.001

Abbreviations: WB: week in which players were born; tB: time of birth; ID: index of discrimination.

Results obtained for the Chi-square analysis comparing the birth-date distribution of finally selected and non-selected soccer players, as well as for the promoted and non-promoted players are reported in Table 3. There was not a significant association between the birth-date distribution and whether or not a player was selected to continue playing to the elite soccer academy in the following season. In addition, there was not a significant association between the birth-date distribution and whether or not a player competed at higher standard of play in their age at the 2018/2019 season.

**Table 3:** Quarterly distribution for the finally selected and non-selected soccer players, as well as for the promoted and non-promoted players.

	Q1	Q2	Q3	Q4	*χ*^2^	df	p
**Selected**	51.1	29.5	13.6	5.7	2.441	3	0.513
**Non-selected**	34.8	39.1	17.4	8.7			
**Promoted**	53.1	28.6	14.3	4.1	1.421	3	0.703
**Non-promoted**	43.5	33.9	14.5	8.1			

Abbreviations: values of the Fisher’s exact test are reported, since the expected frequency of two categories were lower than 5.

### Physical conditioning

The anthropometry, body composition, somatotype and physical performance differences between selected and non-selected soccer players in along each age-category are shown in Table 4. Regarding to U14 age-category, selected players had less fat in sum skinfolds (t_36_ = -2.55, p < 0.05, d = 1.01), showed less percentage of fat (t_36_ = -2.54, p < 0.05, d = 1.01) and higher percentage of muscle (t_36_ = 2.38, p < 0.05, d = 0.95), and presented lower endomorphy (t_36_ = -2.43, p < 0.05, d = 0.97) and mesomorphy (t_36_ = -2.15, p < 0.05, d = 0.85) components compared with non-selected ones. In respect to the U16 age-category, selected players performed better sprint test at 10 (t_29_ = -4.25, p < 0.01, d = 2.07) and 30 m (t_29_ = -2.81, p < 0.01, d = 1.37) distances compared with non-selected ones. No significant differences (p > 0.05) were found for the U18 age-category between selected and non-selected players.

**Table 4:** Anthropometry, body composition, somatotype and physical performance of the selected and non-selected soccer players in along each age-category.

	U14			U16			U18			All		
	Selected	Non-selected	d	Selected	Non-selected	d	Selected	Non-selected	d	Selected	Non-selected	d
**Anthropometry and body composition**												
**Body mass (kg)**	50.69±10.15	51.29±4.31	0.06	62.72±8.17	64.55±3.05	0.24	70.34±7.30	74.78±7.68	0.60	60.74±11.75	63.45±11.56	0.23
**Height (cm)**	162.0±9.4	162.0±6.0	0.04	170.9±6.3	171.6±7.5	0.11	176.8±4.9	180.2±7.2	0.61	169.5±9.4	171.1±10.4	0.17
**Sum skinfolds (mm)**	46.57±10.02*	61.50±26.44	1.01	52.65±15.91	63.67±10.33	0.72	53.52±20.01	56.38±8.83	0.16	50.78±15.70*	60.23±17.15	0.59
**Thigh length (cm)**	41.12±2.89	42.50±2.48	0.49	44.02±6.39	43.33±1.94	0.12	45.82±2.70	46.44±2.47	0.27	43.50±4.78	44.16±2.86	0.15
**Biestyloid at the wrist (cm)**	5.07±0.30	5.16±0.32	0.31	5.41±0.33	5.43±0.29	0.08	5.54±0.30*	5.80±0.22	0.89	5.33±0.37	5.47±0.38	0.38
**Fat (%)**	7.48±1.06*	9.05±2.79	1.01	8.12±1.66	9.27±1.09	0.72	8.21±2.10	8.50±0.94	0.15	7.92±1.64*	8.91±1.81	0.59
**Muscle (%)**	50.15±1.54*	48.50±2.42	0.95	51.03±1.68	50.10±1.70	0.55	52.60±4.11	50.96±1.64	0.44	51.18±2.75*	49.83±2.17	0.51
**Somatotype**												
**Endomorphy**	1.90±0.42*	2.47±1.04	0.97	2.35±0.73	2.83±0.68	0.66	2.16±0.66	2.28±0.34	0.19	2.14±0.64*	2.49±0–75	0.55
**Mesomorphy**	3.03±0.70*	3.67±0.97	0.85	3.24±0.98	3.20±1.33	0.04	3.06±1.66	3.33±0.81	0.18	3.11±1.14	3.42±1.00	0.28
**Ectomorphy**	3.62±0.76	3.28±0.75	0.45	2.99±0.92	2.73±0.93	0.29	2.84±0.74	2.76±0.68	0.11	3.17±0.87	2.94±0.79	0.26
**Physical performance**												
**Abalakov (cm)**	35.98±4.66	36.70±44.40	0.49	40.95±3.99	40.40±3.23	0.14	42.39±3.54	41.81±4.13	0.16	39.71±4.91	38.62±5.35	0.22
**D-Abalakov (cm)**	30.43±3.75	29.86±5.29	0.14	34.80±4.12	33.20±3.39	0.40	35.63±4.05	35.01±4.99	0.14	33.57±4.56	32.76±5.07	0.17
**Sprint 10 m (s)**	1.92±0.10	1.92±0.11	0.03	1.82±0.06*	1.95±0.09	2.07	1.81±0.06	1.79±0.04	0.30	1.85±0.09	1.88±0.11	0.32
**Sprint 30 m (s)**	4.67±0.27	4.70±0.25	0.13	4.33±0.14*	4.52±0.13	1.37	4.25±0.16	4.26±0.09	0.07	4.42±0.27	4.49±0.26	0.26
**Flexibility (cm)**	8.93±5.19	8.75±5.85	0.03	9.48±4.89	7.00±9.67	0.43	11.15±8.95	9.50±9.70	0.18	9.82±6.53	8.62±8.04	0.18

Abbreviations: U14 = under 14 age-category; U16 = under 16 age-category; U18 = under 18 age-category.

* level of signification was set at p < 0.05.

Table 5 shows the differences in anthropometry, body composition, somatotype and physical performance between promoted and non-promoted soccer players in along each age-category. No significant differences (p > 0.05) were found between promoted and non-promoted soccer players in along each age-category on anthropometry, body composition, somatotype and physical performance measures.

**Table 5:** Anthropometry, body composition, somatotype and physical performance of the promoted and non-promoted soccer players in along each age-category.

	U14			U16			U18			All		
	Promoted	Non-promoted	d	Promoted	Non-promoted	d	Promoted	Non-promoted	d	Promoted	Non-promoted	d
**Anthropometry and body composition**												
**Body mass (kg)**	50.87±9.64	50.71±8.69	0.02	65.00±5.05	62.07±8.29	0.39	72.53±8.33	70.86±7.22	0.22	59.55±12.45	62.62±10.92	0.26
**Height (cm)**	161.9±9.4	161.9±7.8	0.01	171.2±4.10	170.6±7.4	0.09	178.4±6.3	177.3±5.3	0.20	168.14±10.23	171.0±8.9	0.30
**Sum skinfolds (mm)**	48.42±12.22	51.93±20.89	0.22	55.92±16.58	54.15±15.23	0.11	49.45±16.19	56.59±18.53	0.40	50.57±14.41	54.52±17.59	0.24
**Thigh length (cm)**	41.21±2.91	41.75±2.77	0.19	43.17±1.71	44.14±6.97	0.17	47.23±2.40	45.23±2.52	0.81	43.12±3.48	43.99±5.04	0.20
**Biestyloid at the wrist (cm)**	5.08±0.29	5.10±0.33	0.05	5.43±0.32	5.40±0.32	0.09	5.68±0.33	5.57±0.29	0.37	5.31±0.39	5.39±0.35	0.21
**Fat (%)**	7.67±1.29	8.05±2.20	0.22	8.45±1.74	8.28±1.59	0.10	7.79±1.71	8.53±1.94	0.40	7.90±1.52	8.32±1.84	0.24
**Muscle (%)**	49.97±1.42	49.52±2.47	0.24	51.25±1.84	50.74±1.70	0.30	52.62±2.76	52.00±4.15	0.17	50.92±2.16	50.91±3.03	0.01
**Somatotype**												
**Endomorphy**	1.94±0.47	2.14±0.85	0.31	2.44±0.70	2.46±0.79	0.02	1.94±0.59	2.31±0.57	0.64	2.07±0.59	2.34±0.73	0.39
**Mesomorphy**	3.14±0.70	3.20±0.97	0.08	3.61±1.02	3.09±0.99	0.52	3.22±1.27	3.08±1.61	0.09	3.28±0.94	3.11±1.23	0.15
**Ectomorphy**	3.55±0.73	3.56±0.83	0.02	2.62±0.82	3.04±0.97	0.51	2.83±1.06	2.82±0.49	0.01	3.14±0.92	3.08±0.83	0.07
**Physical performance**												
**Abalakov (cm)**	36.16±4.45	34.32±4.94	0.40	41.35±5.44	40.79±3.20	0.10	42.56±3.86	42.07±3.56	0.13	38.99±5.32	39.94±4.71	0.19
**D-Abalakov (cm)**	31.16±3.56	28.69±4.50	0.63	35.14±3.58	34.24±4.14	0.25	35.92±4.07	35.24±4.37	0.16	33.29±4.28	33.47±4.91	0.04
**Sprint 10 m (s)**	1.92±0.10	1.91±0.10	0.07	1.82±0.06	1.84±0.08	0.22	1.82±0.06	1.80±0.05	0.33	1.87±0.10	1.84±0.09	0.34
**Sprint 30 m (s)**	4.68±0.25	4.68±0.28	0.00	4.35±0.13	4.36±0.17	0.09	4.25±0.17	4.25±0.13	0.05	4.49±0.29	4.39±0.25	0.40
**Flexibility (cm)**	9.21±5.76	8.31±4.33	0.17	8.00±5.42	9.88±5.83	0.33	9.92±7.61	11.27±9.88	0.15	9.13±6.14	10.05±7.34	0.14

Abbreviations: U14 = under 14 age-category; U16 = under 16 age-category; U18 = under 18 age-category.

The aim of this study was to analyze the influence of the RAE and physical conditioning features (i.e., anthropometry, body composition, somatotype, and physical performance) on the selection and promotion processes in an elite soccer academy. This is the first study including an analysis of the selection and promotion processes of elite youth soccer players, in addition to their relationship with the RAE. The main results showed an effect of RAE on the birth-date distribution of players at U14 and U16 categories, as well as for the overall dataset. However, neither an association was observed between the effect of RAE and players’ selection process, or between RAE and the chance of a player to promote to the higher standard of play in their age. In addition, less fat in sum skinfolds, less percentage of fat, higher percentage of muscle and lower endomorphy and mesomorphy components were showed in U14 players who were selected to continue in the club the following season. Likewise, better sprint performance was demonstrated in selected U16 players, when compared to non-selected players. On the other side, no significant differences on anthropometry, body composition, somatotype and physical performance were found between promoted and non-promoted players.

### Relative age effect (RAE)

The RAE has been well-documented in soccer for the past two decades [1,3,15], with special focus on the Spanish population [2,6,38–41]. Our results further support the notion that players born early in the year are more likely to be selected to play for an elite soccer academy. Interestingly, in line with evidence showing that RAE decreased as age increased [35,38,39], we found an effect of RAE for the U14 and U16 age-categories, although we failed to find this effect for the U18 category. This is in contrast with results reported by Mujika et al. [2], who proposed that the effect of RAE is independent of players’ age. Authors found an over-representation of players born early in the year for senior (Athletic Club Bilbao from *La Liga*), elite youth (U11-U18, third and second teams of the reported club), regional youth (U11-U14), and school youth (U10-U11) categories. These differences might be explained by this soccer club philosophy, which determines its talent development program, since it is based on exclusively drafting players that were either born or grown up in a limited geographical area (Euskal Herria). Thus, further work is needed in order to fully understand factors determining RAE, although previous meta-analysis from Cobley, Baker, Wattie, and McKenna [42] detected age-category and skill level as main factors.

### Physical conditioning

For professional soccer clubs, being successful at selecting youth players for their academies is a priority in order to maintain their sporting and financial status [18]. Considering all players of a soccer academy, Gil et al. [9] observed lower percentage of fat, similar body components and better sprint performance in selected rather than non-selected players. To optimize the evaluation of the selection process, we decided to distinguish players according to their age. In this respect, while our results showed that players from the U14 age-category who were selected to continue playing for the soccer elite academy had lower fat in sum skinfolds, lower percentage of fat, higher percentage of muscle and lower endomorphy and mesomorphy components; other authors did not find differences for anthropometric and body composition measures when comparing selected and non-selected players [19]. Regarding the U16 age-category, the selected players in our study did not show differences in anthropometric and body composition characteristics, which is in line with previous reports from Bidaurrazaga-Letona et al. [19]. In addition, we observed better sprinting capacity at 10 and 30 m distances in selected players, whereas Bidaurrazaga-Letona et al. [19] did not find differences in the values of the physical fitness test. These differences in both age-categories (i.e.; U14 and U16) could be explained by the skill level of players recruited for each study. Whereas players involved in our investigation belonged to a club competing at international level, players from the aforementioned study competed at national level. Finally, we did not find differences for any of the analyzed anthropometric and physical characteristics between selected and non-selected players for the U18 age-category. A plausible explanation for this finding is that at this age soccer-specific skills are the crucial factor determining the likelihood of a player to continue in the club, rather than other secondary anthropometric characteristics and physical capacities [43].

How successful players from an elite soccer academy are in achieving the top soccer professional levels allows to measure the effectiveness of the academy’s talent identification programs [17,21,22]. In this sense, some investigations have demonstrated that sprint and jump performances and aerobic endurance capacity were not pertinent traits to promote a player to the top level of soccer [17,22,24]. Otherwise, Gonaus and Muller [23] reported greater sprint and jump performances in players from U14 and U15 age-categories who subsequently had been drafted at least two times into a youth (U18 to U21) National team. Nevertheless, the success of a talent development program should not only be evaluated by the promotion to professionalism but also by the internal promotion (i.e., selection process) of the youth soccer players within the club. Hence, Deprez et al. [11] analyzed the relevant characteristics of those players who continued playing for the same soccer club two years after the assessments of their physical condition. These authors showed that U14 promoted players presented higher weight and better sprint and jump performances, although they did not find significant differences in anthropometry and physical performance for U16 and U18 age-categories. In order to deepen the study of the promotion process, we decided to analyze the standard of play of each soccer player five years after data collection, attending to their current age-category. Results showed no significant differences in anthropometry, body composition, somatotype and physical performance at different age-categories (i.e., U14, U16 and U18) between promoted and non-promoted players. Therefore, it seems that soccer academies would need to reorient their talent identification programs to make sure that players identified as talented have the potential to promote to higher levels of play within their sport career.

### Association RAE with selection and promotion processes

We did not find an association between RAE and the selection process. Thus, being selected to continue playing at an elite soccer academy was not determined by whether a player was born early or late within a year. When combined with data found for the physical conditioning tests, our results then suggest that coaches from elite soccer academies focused on players’ physical conditioning in order to decide whether they continue playing for the club, specifically in U14 and U16 age-categories. Since players born early in a year show greater physical conditioning compared to players born late in a year [4], it seems reasonable to suggest that RAE is determined by the preference of coaches to recruit and select players showing greater physical capacities.

In addition, we did not find an association between RAE and the promotion process. Thus, the chances of a player of being promoted to the higher standard of play for his age-category was not determined by whether a player was born early or late within a year. When combined with data found for the physical conditioning tests, our results showed that the promotion process was not determined either by the time of birth or the physical condition of a player. Therefore, the promotion process might be determined by other factors, such as soccer-specific skills, as previously proposed by Vaeyens et al. [18].

The aforementioned findings lead us to conclude that elite soccer academies prioritize the short-term success of their teams (i.e., winning youth regional and national tournaments) in all age-categories, instead of the internal promotion of players to higher standard of play within the club. In this sense, a new paradigm for talent identification, selection, and promotion in soccer should be stablished, in order to attend not only to players’ anthropometric and physical characteristics, but also to other relevant indicators such as soccer-specific skills and contextual factors. Thus, future lines of research should address whether soccer-specific skills are a crucial factor determining the promotion of a player to the higher standard of play for his age-category. In addition, it would be of great interest to analyze the tactical or decision-making aspects during the development of training tasks (i.e., small sided games) or official match-play and their possible influence on the promotion at the high competitive-level.

## Conclusions

In this study, an effect of RAE on the birth-date distribution was shown for players at U14 and U16 categories, as well as for the overall dataset. There were not significant associations between the birth-date distribution and whether or not a player was selected to continue playing to the elite soccer academy in the following season, or a player competed or not at higher standard of play in their age at the 2018/2019 season. Thus, considering the lack of association observed between the effect of RAE and players’ selection and promotion processes, it would be necessary a new paradigm which improve the identification of talented soccer players attending not only to the anthropometric and physical characteristics but also to the soccer-specific skills and contextual factors. In addition, given that, on one hand, less fat in sum skinfolds, less percentage of fat, higher percentage of muscle and lower endomorphy and mesomorphy components were observed in U14 players who were selected to continue in the club the following season, and better sprint performance was found for selected U16 players, when compared to non-selected players, and on the other hand, promoted players did not present better anthropometry, body composition, somatotype and physical performance at different age-categories (i.e., U14, U16 and U18) than non-promoted players, soccer academies would need to reorient their talent identification programs to make sure that players identified as talented have the potential to promote to higher levels of play within their sport career considering the specific characteristics of each soccer academy.

## Supporting information

S1 File Manuscript dataset. (XLSX)
